# Pre-test probability estimation of coronary artery disease can be improved by adding an acoustic-based risk score

**DOI:** 10.1016/j.ijcha.2025.101672

**Published:** 2025-04-01

**Authors:** Louise H Bjerking, Samuel E Schmidt, Kim W Skak-Hansen, Simon Winther, Morten Böttcher, Søren Galatius, Eva Prescott

**Affiliations:** a Department of Cardiology, Copenhagen University Hospital Bispebjerg and Frederiksberg, Denmark; b Department of Health Science and Technology, Biomedical Engineering & Informatics, Aalborg University, Denmark; c Department of Cardiology, Gødstrup Hospital, Herning, Denmark

**Keywords:** Chronic coronary syndrome, Coronary artery disease, Heart sound, Pre-test probability, Risk stratification

## Abstract

**Background:**

The American Heart Association/American College of Cardiology (AHA/ACC) 2021 Chest Pain Guidelines introduced a new pre-test probability (PTP) model for obstructive coronary artery disease (CAD). The model recommends a 15 % risk cut-off for referral for further testing. Whether addition of a risk score measured from acoustic detection of coronary turbulence obtained by the noninvasive device CADScor®System (CAD-score) improves the AHA/ACC-PTP capability to assign the correct risk category has not been tested.

**Methods:**

Patients with symptoms suggestive of CAD referred for coronary CT angiography and undergoing a same-day CAD-score were included. PTP was calculated based on sex, age, and symptoms. All patients with suspected stenosis on CT angiography were referred for invasive angiography. A CAD-score ≤ 20 was used as cut-off for low likelihood of CAD.

**Results:**

The study population consisted of 2874 patients (47 % women, median age [IQR] 58 [52–65] years). PTP categorized 2044 (71 %) of patients as > 15 % amongst whom 387 (18.9 %) were re-classified to low likelihood by a CAD-score ≤ 20. In patients aged < 70 without hypertension, 37 % were re-classified to low probability. Of the 830 patients with low PTP ≤ 15 %, 68.7 % had a CAD-score ≤ 20 indicating a deferred testing strategy.

**Conclusion:**

Adding an acoustic-based CAD-score to the PTP in patients with AHA/ACC defined-PTP > 15 % risk can reduce the number of diagnostic tests by overall 19 %, and 37 % in subgroups, and may support cost-effective clinical decision-making. Moreover, CAD-score may aid risk stratification in patients, particularly with AHA/ACC-PTP ≤ 15 %.

## 1 Introduction

Symptoms suggestive of chronic coronary disease in patients without known coronary artery disease (CAD) are a common reason for outpatient cardiac diagnostic testing. However, the diagnostic yield has proven to be low, and recent trials indicate limited benefit of an invasive strategy in many patients with obstructive CAD [1], [2], [3]. Hence, cost-effective, and simple ways to identify low-risk patients with little benefit from advanced diagnostic testing strategies are warranted.

In 2021, the American Heart Association (AHA)/American College of Cardiology (ACC)/American Society of Echocardiography/American College of Chest Physicians/Society for Academic Emergency Medicine/Society of Cardiovascular Computed Tomography/Society for Cardiovascular Magnetic Resonance Guideline for Evaluation and Diagnosis of Chest Pain was published [4]. The guidelines introduced an updated pre-test probability (PTP) table based on previously published PTP models [5], [6] to guide the clinical investigation pathway for patients with stable chest pain and no known CAD. In contrast to the European Society of Cardiology and the National Institute for Health & Care Excellence, the PTP does not subdivide chest pain into typical, atypical, and non-anginal chest pain [4], [7], [8], [9]. Similarly to the European Society of Cardiology 2019 guidelines, the PTP was generally downgraded in the AHA/ACC 2021 Chest Pain guidelines [4], [8].

According to the AHA/ACC 2021 Chest Pain guideline, patients without known CAD and an intermediate-high likelihood of CAD (PTP > 15 %) should undergo non-invasive testing as a first-line test to guide further treatment [4]. Patients with PTP ≤ 15 % have a low likelihood of obstructive CAD and should not routinely undergo further diagnostic tests. In selected cases, exercise ECG or coronary artery calcium score (CACS) are suggested for further risk stratification, but no specific guidance on how to identify those patients is provided in the guidelines [4].

A potentially fast, low-cost, non-invasive, and radiation-free way to identify patients with low likelihood of CAD is to add an acoustic-based probability score [10], [11], [12], [13], [14]. One device for assessment of an acoustic probability scores is the CADScor®System (Acarix A/S). It derives acoustic information obtained by a microphone fixed by a patch to the chest, which, integrated with clinical information on sex, age, and hypertension in an algorithm, results in a score ranging from 0 to 99 (the CAD-score) [15]. The device has been cleared by the U.S. Food and Drug Administration [16] and has shown good rule-out capabilities. In one previous study including patients suspected of obstructive CAD undergoing coronary computed tomography angiography (CCTA), a negative predictive value (NPV) of 97.2 % and sensitivity of 88.7 % was seen using a cut-off CAD-score ≤ 20 [14], [17]. Moreover, a low CAD-score ≤ 20 was associated with a better prognosis [18].

In this study, we examine if adding a CAD-score to the PTP has the potential to 1) correctly re-classify patients from intermediate-high likelihood of CAD (PTP > 15 %) to low likelihood and thereby allow rule-out and defer diagnostic testing, and 2) improve risk stratification in patients with low likelihood of CAD (PTP ≤ 15 %) and thereby support the rule-out strategy.

## 2 Methods

### 2.1 Study population

**Fig. 1:**
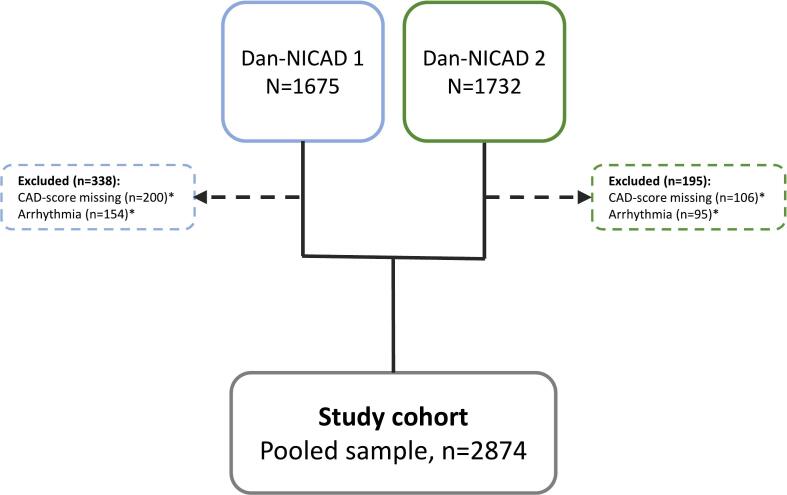
Flowchart. Flowchart showing selection of study cohort. *Patients can be excluded due to both arrhythmia and missing CAD-score. CAD, coronary artery disease; CCTA, Coronary computed tomography angiography; FFR, fractional flow reserve; ICA, invasive coronary angiography.

The present study uses data pooled from the Danish study of Non-Invasive testing in Coronary Artery Disease (Dan-NICAD) 1 (NCT02264717) and Dan-NICAD 2 (NCT03481712) study cohorts. The design of the Dan-NICAD 1 and 2 study has previously been described in detail [19], [20]. In brief, Dan-NICAD 1 and Dan-NICAD 2 enrolled 1675 and 1732 consecutive patients with symptoms suggestive of stable CAD referred for CCTA. In both studies, a CAD-score measurement was made with the non-invasive acoustic device CADScor®System in relation to CCTA. The present study includes data on all patients with symptoms suggestive of chronic coronary disease and a successfully obtained CAD-score (n = 2874) (Fig. 1).

### 2.2 Ethics

The Dan-NICAD 1 and Dan-NICAD 2 study was conducted in accordance with the Declaration of Helsinki, and all included patients signed a written informed consent form. The study was approved by The Central Denmark Region Committees on Health Research Ethics, The Danish Medicines Agency, and The Danish Data Protection Agency.

### 2.3 Hemodynamic obstructive CAD

All patients with suspected significant stenosis at CCTA were referred to invasive coronary angiography. Fractional flow reserve was performed in patients with 30–90 % visually estimated diameter stenosis in vessels > 2.0 mm if technically possible. Hemodynamic obstructive CAD was defined from angiography as > 90 % luminal diameter stenosis (visual assessment) or fractional flow reserve (FFR) < 0.80. If FFR was not done in patients with 30–90 % diameter stenosis, hemodynamic obstructive CAD was defined as ≥ 50 % luminal diameter stenosis reduction assessed by quantitative angiography.

### 2.4 CAD-score

The CAD-score is an acoustic-based probability score for obstructive CAD measured by a 10-minute recording of heart sounds performed with the non-invasive acoustic device CADScor®System [14]. Patients lie supine with the device attached by a patch placed on the chest. A microphone in the device records heart sounds at the left fourth intercostal space during a three-minute period in which the patients are advised to hold their breath four times for eight seconds [14]. Using a linear discriminant function, eight acoustic features obtained in the recording are combined into an acoustic score [17]. This acoustic score is combined with sex, age, and history of hypertension using logistic regression, which derives the CAD-score [17]. A CAD-score ≤ 20 indicates a low likelihood of CAD in previous studies [15].

The Food and Drug Administration cleared algorithm (version 3.2-US) used in this study was developed for the US market.

### 2.5 Pre-test probability

We calculated PTP according to the AHA/ACC 2021 Chest Pain guideline from age, sex and symptom (chest pain or dyspnea) [4]. Chest pain was defined as any type of angina pectoris (typical, atypical, or non-specific chest pain). The AHA/ACC-PTP table categorizes chest pain patients in intervals of PTP delineated by a “≤” symbol. We chose to use the highest possible PTP for each interval e.g., for a male patient aged 55 with chest pain categorized as PTP ≤ 32 we used the value 32.

### 2.6 Statistics

Discrete data are reported as counts and percentages, and continuous variables as mean and standard deviation or median and interquartile range (IQR). The PTP and CAD-score variables were transformed into dichotomous variables; PTP ≤ 15 %/PTP > 15 % and CAD-score ≤ 20/CAD-score > 20. By dividing patients with PTP > 15 % into two groups (CAD-score ≤ 20 vs. > 20), the reclassification of patients with PTP > 15 % to low probability of CAD was calculated in all patients and subgroups (age, sex, hypertension and PTP). The ability of the CAD-score to risk stratify was explored by dividing patients with PTP ≤ 15 % into CAD-score ≤ 20 vs. > 20. The diagnostic performance of the CAD-score compared to the PTP was evaluated by calculating sensitivity, specificity, negative predictive value, positive predictive value, negative likelihood ratio, and positive likelihood ratio at the cut-off CAD-score 20 and PTP 15 % following the cut-off for low vs. intermediate-high likelihood for CAD. The area under the receiver-operating curve (AUC) was calculated for the continuous CAD-score and PTP variables individually and compared with the DeLong methods [21].

The CAD-score US algorithm (version 3.2-US) used for patient stratification in this study was developed in a training set selected from the proprietary Acarix heart sound database. Details of the database have been described by Schmidt et al. [17]. The training set included recordings from 1,177 patients; 259 from a study by Winther et al. [15], 342 from the DanRisk 5-year follow-up study [22] and 576 subjects from the Dan-NICAD 1 cohort which is included in the current study. The remaining patients from Dan-NICAD 1 were used as a test set for validation and the Dan-NICAD 2 cohort for external validation (supplementary Table S1). Since the diagnostic performance of the algorithm was comparable across training and test set, the analyzes in this current study were conducted in a pooled data set of the full Dan-NICAD 1 and 2 cohort.

All statistical analyses were performed with the statistical software R, version 4.1.0 or Matlab version A2022. All tests had a two-sided significance level of 0.05.

## 3 Results

In total, 2874 patients (47.7 % women) with successfully obtained CAD-score were included in the study (Fig. 1). Hemodynamically obstructive CAD was diagnosed in 322 patients (11.2 %). Baseline characteristics for all patients are shown in Table 1. Patients had a median [IQR] age of 58 [52–65] years, median CAD-score was 22 [13–32] and 66.7 % had CAD-score > 20. Median PTP was 22 % [13–32 %], and 71 % of the study population presented with PTP > 15 %. Compared to patients with low PTP ≤ 15 %, patients with intermediate-high PTP > 15 % were older and had a higher burden of risk factors as well as a higher CAD-score (16 [IQR: 12, 22] vs. 29 [22, 37] p < 0.001) and more obstructive CAD (4.8 % vs. 13.8 %, p < 0.001). Baseline characteristics according to CAD-score and PTP are shown in Supplementary Tables S2 and S3. Higher age and more hypertension were present in patients with high CAD-score independently of PTP.

**Table 1:** Baseline characteristics.

		**PTP**		
	**All** **n = 2874**	**≤ 15 %** **n = 830**	**> 15 %** **n = 2044**	***p-value***
Age, median [IQR]	58.5 [52, 65.3]	53 [49, 58]	61.6 [54, 67]	<0.001
Sex, male (%)	1502 (52.3)	21 (2.5)	1481 (72.5)	<0.001
Symptoms (%)				<0.001
Typical chest pain	734 (25.5)	183 (22.0)	551 (27.0)	
Atypical chest pain	1128 (39.2)	290 (34.9)	838 (41.0)	
Nonspecific	654 (22.8)	165 (19.9)	489 (23.9)	
Dyspnø	358 (12.5)	192 (23.1)	166 (8.12)	
Hypertention (%)	1208 (42.0)	269 (32.4)	939 (45.9)	<0.001
Family history of CAD	1030 (35.8)	348 (41.9)	682 (33.4)	<0.001
Diabetes mellitus (%)	157 (5.5)	45 (5.4)	112 (5.5)	1.000
BMI, mean (SD)	27.1 (4.2)	26.7 (4.7)	27.3 (3.99)	<0.001
Systolic blood pressure, mean (SD)	137.7 (18.3)	131.7 (18.6)	140.6 (17.65)	<0.001
Diastolic blood pressure, mean (SD)	83.6 (10.4)	81.5 (10.6)	84.4 (10.2)	<0.001
Heart rate, mean (SD)	62.4 (9.9)	63.5 (10.1)	61.9 (9.8)	<0.001
EF, mean (SD)	59.7 (3.7)	60 (3.8)	59.6 (3.6)	0.011
AHA/ACC-PTP, median [IQR]	22 [13, 32]	13 [10], [13]	32 [22, 44]	<0.001
CAD-score, median [IQR]	25 [17, 34]	16 [12], [21]	29 [22, 37]	<0.001
CAD-score > 20 (%)	1917 (66.7)	260 (31.3)	1657 (81.1)	<0.001
Hemodynamic obstructive CAD (%)	2552 (88.8)	790 (95.2)	1762 (86.2)	<0.001
CACS, median [IQR]	3 [0, 93]	0 [0, 10]	17 [0, 152]	<0.001
CACS groups (%)				<0.001
CACS = 0	1336 (46.5)	574 (69.2)	762 (37.4)	
CACS 1–399	1223 (42.7)	220 (26.5)	1003 (49.2)	
CACS ≥ 400	309 (10.8)	35 (4.2)	274 (13.4)	
LAD stenosis (%)	224 (7.8)	21 (2.5)	203 (9.9)	<0.001
RCA stenosis (%)	150 (5.2)	16 (1.9)	134 (6.6)	<0.001
CX stenosis (%)	136 (4.7)	14 (1.7)	122 (6.0)	<0.001
LM stenosis (%)	14 (0.5)	1 (0.1)	13 (0.6)	0.133

Baseline characteristics of the total population and by PTP ≤ 15 % vs. PTP > 15 %. ACC, American College of Cardiology; AHA, American Heart Association; BMI, body mass index; CACS, coronary artery calcium score, CAD, coronary artery disease; CVD, cardiovascular disease; CX, circumflex artery; IQR, interquartile range; LAD, left anterior descending artery, LM, left main; LVEF, left ventricular ejection fraction; PTP, pre-test probability; QCA, qualitative comparative analysis; RCA, right coronary artery; SD, standard deviation.

### 3.1 CAD-score in patients with intermediate-high pre-test probability of obstructive CAD

**Fig. 2:**
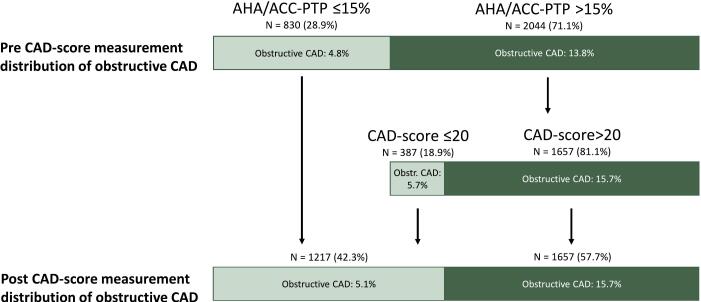
Reclassification in patients with AHA/ACC-PTP > 15 %. Distribution of obstructive CAD before and after reclassification of patients with intermediate-high PTP > 15 % by CAD-score. Light green bars are patients with low likelihood of CAD (rule-out) and dark green bars are patients with intermediate-high likelihood of CAD. Obstructive CAD is defined as hemodynamic obstructive CAD on ICA. ACC, American College of Cardiology; AHA, America Heart Association; CAD, Coronary artery disease; ICA, invasive coronary angiography; PTP, Pre-test probability.

Fig. 2 shows the re-classification of patients with PTP > 15 % by CAD-score. Addition of a CAD-score to the 2044 (71.1 % of all patients) with PTP > 15 %, re-classified 387 (18.9 %) patients to low likelihood. In these patients, the prevalence of hemodynamically obstructive CAD was significantly lower than in the 773 patients with PTP > 15 % and CAD-score > 20 (5.7 % vs. 15.7 %, p < 0.001). After adding the CAD-score to PTP the proportion classified as low-likelihood increased from 830 (28.9 %) to 1217 (42.3 %) patients. Concomitantly, the prevalence of potentially undiagnosed hemodynamically obstructive CAD after re-classifying to low risk for CAD-score ≤ 20 increased slightly from 4.8 % (n = 40) to 5.1 % (n = 62). Net reclassification improvement was 0.129 for this classification scheme.

**Fig. 3:**
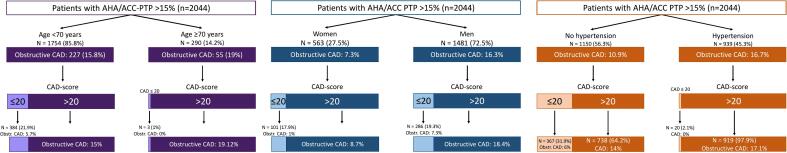
Reclassification in AHA/ACC-PTP > 15 % subgroups. Proportion of patients with CAD-score ≤ 20 (rule-out) and CAD-score > 20 (rule-in) in patients with intermediate-high likelihood of CAD (PTP > 15 %) for A) patients aged < 70 vs. ≥ 70 years, B) women vs. men, C) patients with no hypertension vs. patients with hypertension, and D) patients with PTP > 15–25 % vs. PTP > 25 %.

To explore whether CAD-score could be used more efficiently in subgroups, we assessed reclassification in subgroups defined by age, hypertension, and sex (Fig. 3).

The ability of the CAD-score to re-classify patients was greatest in both male and female patients without hypertension and aged below 70. When adding a CAD-score in the 979 patients below age 70 without hypertension, 36.4 % (prevalence of obstructive CAD: 6 %) had a CAD-score ≤ 20 and indicating re-classification to low probability (Supplementary Fig. S1). Net reclassification improvement was 0.190 for this classification scheme. Conversely, the CAD-score was not able to re-classify any of the 164 patients aged ≥ 70 with hypertension, and only 23 (2.2 %) of the 1065 patients with hypertension *or* age ≥ 70.

### 3.2 **Patients with low pre-test probability of obstructive CAD (PTP** ≤ **15 %)**

**Fig. 4:**
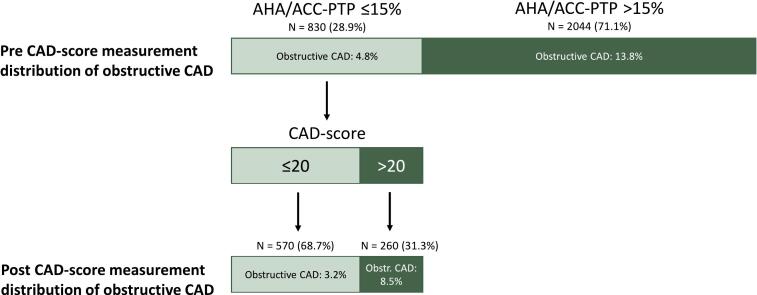
Reclassification in patients with AHA/PTP ≤ 15 %. Distribution of obstructive CAD before and after risk stratification of patients with low PTP ≤ 15 % by CAD-score. Light green bars are patients with low likelihood of CAD (rule-out) and dark green bars are patients with intermediate-high likelihood of CAD. Obstructive CAD is defined as hemodynamic obstructive CAD on ICA. ACC, American College of Cardiology; AHA, America Heart Association; CAD, Coronary artery disease; ICA, invasive coronary angiography; PTP, Pre-test probability.

CAD-score application in the 830 (28.9 %) patients with PTP ≤ 15 % documents the ability of improved risk stratification for obstructive CAD in this group. Of the 570 patients with PTP ≤ 15 % and CAD-score ≤ 20 (Fig. 4), only 3.2 % had hemodynamically obstructive CAD, while 8.5 % of the 260 patients with PTP ≤ 15 % and CAD-score > 20 had obstructive CAD. Net reclassification improvement was 0.249 for this classification scheme.

### 3.3 Suggested flowchart for clinical use

**Fig. 5:**
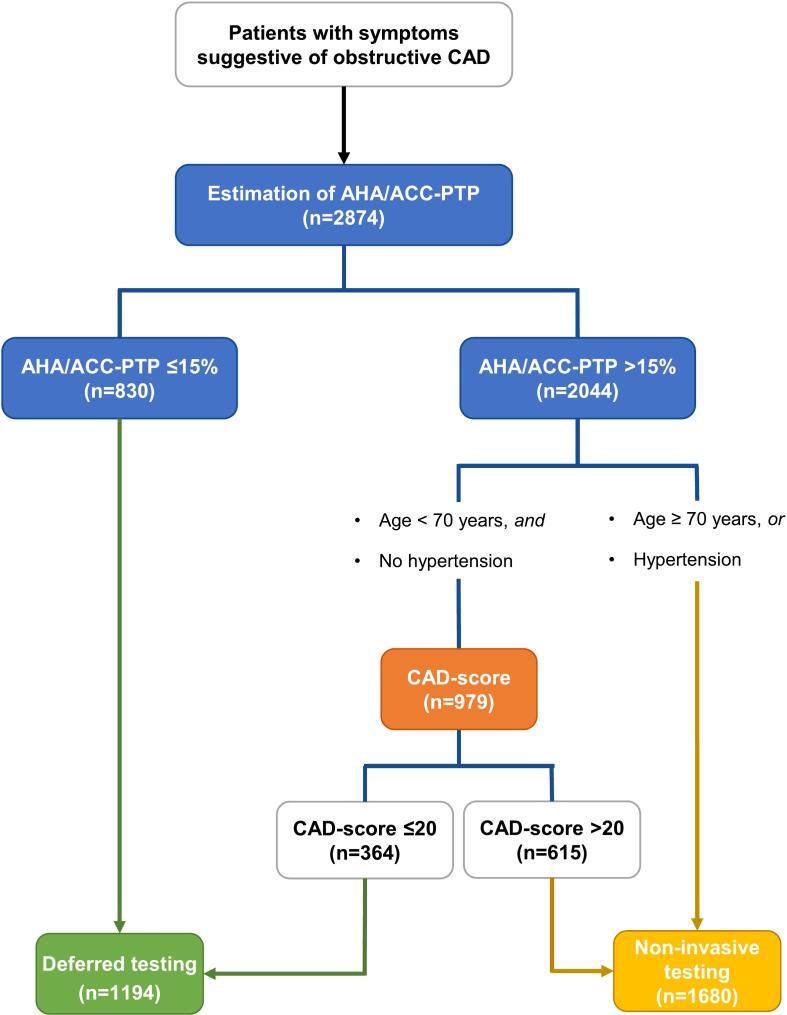
Suggested flowchart for clinical use. Suggestion for implementation of CAD-score as an add-on risk score to PTP in patients without hypertension and age below 70 years. ACC, American College of Cardiology; AHA, American Heart Association; CAD, coronary artery disease; NIT, non-invasive test; PTP, pre-test probability.

Based on subgroup analysis showing limited diagnostic value of the CAD-score in patients ≥ 70 years and with hypertension, we suggest one focused strategy for clinical use of CAD-score illustrated in a flowchart CAD-scoring only patients with PTP > 15 %, low age and without hypertension (Fig. 5).

### 3.4 CAD-score versus AHA/ACC-PTP for risk prediction

The CAD-score alone performed similarly to the PTP alone (AUC of the CAD-score was 70.0 % [95 % CI 66.7–73.3] versus AUC of the PTP 69.9 % [66.6–73.3], p = 0.094). Diagnostic performances of the CAD-score and PTP are summarized in Supplementary Table S4. A re-classification table is shown in Supplementary Table S5. The net reclassification index was 0.0498. Study-specific results showing robustness between the two studies are available in Supplementary Table S6.

## 4 Discussion

### 4.1 Main findings

This study, using pooled data from two large cohorts of consecutive patients with symptoms suggestive of CAD and no known CAD showed that adding a CAD-score to the PTP in patients with an intermediate-high likelihood (PTP > 15 %) of CAD could re-classify more than one out of six to low likelihood indicating no further diagnostic tests was needed. If applied only to younger patients with no hypertension, the CAD-score may downgrade as many as one out of three patients with intermediate-high likelihood to low likelihood without increasing the post-CAD-score probability of hemodynamically obstructive CAD. Furthermore, among those with a low likelihood (PTP ≤ 15 %), the application of a CAD-score stratified the patients into very low likelihood (69 %) and low likelihood (31 %). Hence, CAD-score may aid in supporting deferring testing in more than two-thirds, and thereby be an alternative to the guidelines suggested CACS or exercise ECG risk stratification. Therefore, using the CAD-score in *addition to* the PTP, may be a supporting tool for reducing the use of inappropriate downstream diagnostic tests, patient exposure to radiation and contrast, incidental findings, unnecessary patient concerns, and time-to-CAD-rule-out.

### 4.2 Intermediate-high pre-test probability of obstructive CAD

The 2021 AHA/ACC Guidelines for Evaluation and Diagnosis of Chest Pain provide an easy way to stratify patients with symptoms suggestive of CAD and no known CAD into two groups, low likelihood or intermediate-high likelihood, by estimating their PTP by sex, age, and presence of either chest pain or dyspnea [4]. Patients with PTP > 15 % are recommended to undergo non-invasive diagnostic tests to guide further treatment [4]. However, this group includes a large majority of patients without obstructive CAD (the CAD prevalence in our cohort was only 13.8 %) referred for unnecessary and costly diagnostic tests. Possible overuse of diagnostic tests in PTP-guided referrals has previously been shown [1], [2]. In our study cohort, when applying the AHA/ACC PTP, 71.1 % of patients had intermediate-high CAD likelihood. Of this patient group, 18.9 % were reclassified to low likelihood by a CAD-score ≤ 20. Notably, the prevalence of obstructive CAD was only 5.7 % in these down-classified patients in contrast to 15.7 % in the remaining 81.1 % of the patients. Hence, CAD-score has the potential to reduce the number of diagnostic tests by at least one-sixth easily and inexpensively in patients initially classified as intermediate-high likelihood. Our findings of the potential of adding a CAD-score to the PTP are consistent with previous studies: In a pooled analysis of 2245 patients, CAD-score had a good rule-out capability with a sensitivity of 88.7 % and a negative predictive value of 97.2 % [23]. In the same cohort, Schmidt et al. showed the potential of downgrading one-third of the patients from intermediate-high to low likelihood using CAD-score in addition to the European Society of Cardiology 2019 PTP model [23]. Furthermore, in our study, if choosing a more focused approach by applying CAD-score only to the 979 patients with no hypertension and age < 70, as many as 37 % could safely be ruled out. Conversely, the CAD-score is of less value in patients over age 70 and in patients with hypertension. Based on our subgroup analysis, we suggested a focused strategy for the use of CAD-score in addition to AHA/ACC-PTP estimation (Fig. 5).

### 4.3 Low pre-test probability of obstructive CAD

According to guidelines, diagnostic testing in the low AHA/ACC-PTP group can be deferred in most patients [4]. An additional class 2b recommendation suggests CACS or exercise ECG as a first-line test for further identifying patients with very low vs. higher likelihood of CAD, however, without any clear guidance of who to apply this to [4]. A CACS of zero has shown very good rule-out capability [24], [25], but this tool is expensive and is not easily available everywhere and it comes with radiation, a high incidental finding rate, and patient anxiety [26]. Alternatively, the inexpensive CAD-score can be performed non-invasively within 10 min in an outpatient clinic and might be used for ruling out patients. In this study, 28.9 % of the patients were classified as low likelihood by PTP ≤ 15 %. Of these, 4.8 % had obstructive CAD. Addition of CAD-score in these low likelihood patients, further risk-stratified the group such that two-thirds of patients had a CAD-score ≤ 20 of whom only 3.2 % had obstructive CAD, and one-third with a CAD-score of > 20 of whom 8.5 % had obstructive CAD. Hence, a fast, low-cost, non-invasive CAD-score measurement may be a tool to support the deferral of further diagnostic testing in patients with low likelihood of obstructive CAD. However, CAD-score measurements on all 830 patients with low PTP ≤ 15 %, still only resulted in identification of 22 patients with hemodynamically obstructive CAD out of 260 patients with CAD-score > 20.

## 5 Limitations

This study has several limitations. First of all, the study does not allow conclusions concerning the potential cost-benefit of adding a CAD-score to PTP. Moreover, our study included only patients with suspected stable CAD referred for CCTA. This may introduce selection bias, as the study cohort does not represent the full variety of individuals undergoing cardiac evaluation for symptoms suggestive of CAD. Specifically, the study does not include patients with very low pre-test probability of CAD who were examined by a cardiologist but never referred for a CCTA, nor those with very high PTP who were referred directly to an invasive coronary angiography. However, CCTA is the recommended first-line and preferred technique for non-invasive evaluation in eligible patients in this catchment area. Furthermore, only participants from Danish hospitals with primarily Caucasian patients were included and the results may not be representative of other populations. Hence, the generalizability of our findings to more ethnically diverse populations is uncertain.

The AHA/ACC guidelines present each PTP for chest pain as an “equal to or smaller than” PTP category. We have chosen to use the maximum value for each PTP category. In a real-life setting the treating physician might have downgraded the PTP based on e.g. absence of cardiac risk factors. However, downgrading risk can be difficult, and the diagnostic strategy may often be defensive with a high referral rate. In this situation, CAD-score may serve as a valuable risk-downgrading tool, potentially improving patient selection for further testing. In terms of real-world applicability, it is worth noting that the CAD-score®System is easy to use and can be applied in a relatively short amount of time. However, a potential limitation is that it requires a quiet environment to ensure accurate assessment, which may not always be achievable in busy clinical settings.

In this study, no clinically consequences were taken to the CAD-score in this study, and assumptions about prognosis or safety concerning the CAD-score rule-out strategy cannot be made. However, the recently published FILTER-SCAD study showed that adding the CAD-score to the European Society of Cardiology PTP was safe compared to PTP alone when selecting patients for a deferred testing strategy [27]. Finally, a subset of the current cohort was used for training the algorithm, meaning that the algorithm might be overfitted to these data. However, no statistical difference was observed in diagnostic performance across the training set and the remaining dataset (Supplementary Table S5).

## 6 Conclusion

This study in patients with suspected CAD shows that a simple, non-invasive, low-cost, acoustic-based CAD-score could potentially reduce the number of non-invasive tests among patients with intermediate-high PTP > 15 % by 19 % and in selected subgroups by 37 %. Moreover, CAD-score applied in patients with low PTP ≤ 15 % improved pre-test probability estimation. Therefore, adding a CAD-score to the PTP in patients with suspected CAD may support the clinical decision-making process, particularly in patients with intermediate-high PTP, by improving diagnostic accuracy.

## Source of funding

None for this study. Acarix provided a grant for the Dan-NICAD 1 and Dan-NICAD 2 study.

## Disclosures

LHB: none, SES: holds significant shares in Acarix, works significantly as Expert Witness for Acarix and received institutional research grants from Acarix, SW: none, KWSH: none, SG, discloses advisory board participation in Astra Zeneca, Boehringer Ingelheim, MSD, EP: none, MB: advisory board participation for NOVO Nordisk, Astra-Zeneca, Novartis, Boehringer Ingelheim, Bayer, Sanofi, and Acarix.

**Acknowledgement of grant support:** None for this study. Acarix provided a grant for the Dan-NICAD 1 and Dan-NICAD 2 study.

## CRediT authorship contribution statement

**Louise H Bjerking:** Writing – review & editing, Writing – original draft, Methodology, Investigation, Formal analysis, Conceptualization. **Samuel E Schmidt:** Writing – review & editing, Supervision, Methodology, Formal analysis, Conceptualization. **Kim W Skak-Hansen:** Writing – review & editing, Supervision, Conceptualization. **Simon Winther:** Writing – review & editing, Supervision, Conceptualization. **Morten Böttcher:** Writing – review & editing, Supervision, Conceptualization. **Søren Galatius:** Writing – review & editing, Supervision, Conceptualization. **Eva Prescott:** Writing – review & editing, Supervision, Conceptualization.

## Declaration of competing interest

The authors declare the following financial interests/personal relationships which may be considered as potential competing interests: [LHB: none, SES: holds significant shares in Acarix, works significantly as Expert Witness for Acarix and received institutional research grants from Acarix, SW: none, KWSH: none, SG, discloses advisory board participation in Astra Zeneca, Boehringer Ingelheim, MSD, EP: none, MB: advisory board participation for NOVO Nordisk, Astra-Zeneca, Novartis, Boehringer Ingelheim, Bayer, Sanofi, and Acarix].
