# Supplementary material for: Pre-test probability estimation of coronary artery disease can be improved by adding an acoustic-based risk score
Source: Int J Cardiol Heart Vasc. 2025 Apr 1;58:101672. doi: 10.1016/j.ijcha.2025.101672 (PMC11999282; doi:10.1016/j.ijcha.2025.101672)
Supplement: Supplementary Data 1 [file mmc1.docx]

**Supplemental Material**

[**Supplemental Material** 1](#_Toc181865443)

[**Supplementary tables** 2](#_Toc181865444)

[Table S1 – Diagnostic performance of the CAD-score at cut-off 20 in patients used for algorithm development (Training set), patients used for validation (Test set) and External Validation Set 2](#_Toc181865445)

[Table S2 – Baseline characteristics of patients with low pre-test probability stratified by high and low CAD-score measurement 3](#_Toc181865446)

[Table S3 – Baseline characteristics of patients with intermediate-high pre-test probability stratified by low and high CAD-score measurement 5](#_Toc181865447)

[Table S4 – Diagnostic performance of the CAD-score at cut-off 20 and AHA/ACC-PTP at cut-off 15% 7](#_Toc181865448)

[Table S5: Reclassification table 8](#_Toc181865449)

[Table S6: Study-specific reclassification in low & high PTP. 10](#_Toc181865450)

[**Supplementary figure and figure legend** 11](#_Toc181865451)

[Figure S1 11](#_Toc181865452)

## **Supplementary tables**

Table S1 – Diagnostic performance of the CAD-score at cut-off 20 in patients used for algorithm development (Training set), patients used for validation (Test set) and External Validation Set

|  | **All** | **Training Set**  **(Dan-NiCAD I)** | **Test Set**  **Dan-NiCAD I)** | **External Validation Set (Dan-NiCAD II)** |
| --- | --- | --- | --- | --- |
| N: Other | 2552 | 467 | 738 | 1347 |
| N: CAD | 322 | 44 | 88 | 190 |
| Prevalence of CAD (p=0.056) | 11.2% | 8.61% | 10.7% | 12.4% |
| AUC (p=0.527) | 70% (66.7-73.3) | 69.2% (60.2-78.1) | 71.1% (64.8-77.4) | 69.1% (64.8-73.5) |
| NPV (p=0.451) | 95.8% (94.4-97%) | 97.4% (94.1-99.2%) | 95.6% (92.6-97.6%) | 95.3% (93-97%) |
| PPV (p=0.2852) | 14.7% (13.2-16.4%) | 12.3% (8.9-16.4%) | 14.1% (11.3-17.3%) | 15.7% (13.6-18.1%) |
| Sensitivity (p=0.735) | 87.6% (83.5-91%) | 88.6% (75.4-96.2%) | 85.2% (76.1-91.9%) | 88.4% (83-92.6%) |
| Specificity (p=0.007) | 35.9% (34.1-37.8%) | 40.5% (36-45.1%) | 38.1% (34.6-41.7%) | 33.2% (30.7-35.8%) |
| Rule out (p=0.002) | 33.3% (31.6-35.1%) | 38% (33.7-42.3%) | 35.6% (32.3-39%) | 30.5% (28.2-32.9%) |
| Rule in (p=0.002) | 66.7% (64.9-68.4%) | 62% (57.7-66.3%) | 64.4% (61-67.7%) | 69.5% (67.1-71.8%) |
| True negative, n | 917 | 189 | 281 | 447 |
| False negative, n | 40 | 5 | 13 | 22 |
| False positive, n | 1635 | 278 | 457 | 900 |
| True positive, n | 282 | 39 | 75 | 168 |
| PLR | 1.367 | 1.489 | 1.376 | 1.323 |
| NLR | 0.3457 | 0.2808 | 0.388 | 0.3489 |

*AUC, NPV, PPV, sensitivity, specificity, PLR, and NLR for CAD-score at cut-off 20 and PTP at cut-off 15%. AUC, area under the receiver-operating curve; NLR, negative likelihood ratio; NPV, negative predictive value; PLR, positive likelihood ratio; PPV, positive predictive value.*

Table S2 – Baseline characteristics of patients with low pre-test probability stratified by high and low CAD-score measurement

|  | CAD-score ≤20 | CAD-score>20 | p-value |
| --- | --- | --- | --- |
| n | 570 | 260 |  |
| Age, median [IQR] | 51.29 [47.00, 56.00] | 58.00 [53.96, 66.00] | <0.001 |
| Sex, male (%) | 14 (2.5) | 7 (2.7) | 1.000 |
| Symptoms (%) |  |  | <0.001 |
| Typical chest pain | 135 (23.7) | 48 (18.5) |  |
| Atypical chest pain | 227 (39.8) | 63 (24.2) |  |
| Nonspecific | 133 (23.3) | 32 (12.3) |  |
| Dyspnea | 75 (13.2) | 117 (45) |  |
| Smoking (%) |  |  | 0.425 |
| Active smoker | 178 (31.2) | 83 (31.9) |  |
| Former smoker | 162 (28.4) | 63 (24.2) |  |
| Never | 230 (40.4) | 114 (43.8) |  |
| Hypertention (%) | 93 (16.3) | 176 (67.7) | <0.001 |
| RELATI = TRUE (%) | 248 (43.5) | 100 (38.5) | 0.197 |
| Diabetes (%) | 27 (4.7) | 18 (6.9) | 0.261 |
| BMI, mean (SD) | 26.78 (4.87) | 26.41 (4.38) | 0.296 |
| Systolic BP mean (SD) | 125.55 (15.50) | 145.04 (17.81) | <0.001 |
| Diastolic BP, mean (SD) | 79.68 (10.20) | 85.60 (10.28) | <0.001 |
| Heart rate, mean (SD) | 63.13 (10.19) | 64.34 (9.88) | 0.111 |
| LVEF, mean (SD) | 60.16 (3.56) | 59.64 (4.20) | 0.066 |
| AHA/ACC-PTP, median [IQR] | 13.00 [10.00, 13.00] | 13.00 [13.00, 13.00] | <0.001 |
| CAD-score, median [IQR] | 13.00 [11.00, 16.00] | 24.00 [22.00, 29.00] | <0.001 |
| Hemodynynamic obstructive CAD (%) | 552 (96.8) | 238 (91.5) | 0.002 |
| CACS, median [IQR] | 0.00 [0.00, 0.00] | 0.00 [0.00, 57.50] | <0.001 |
| CACS groups (%) |  |  | <0.001 |
| 1 | 436 (76.6) | 138 (53.1) |  |
| 2 | 124 (21.8) | 96 (36.9) |  |
| 3 | 9 (1.6) | 26 (10.0) |  |
| LAD stenosis (%) | 9 (1.6) | 12 (4.6) | 0.019 |
| RCA stenosis (%) | 8 (1.4) | 8 (3.1) | 0.176 |
| CX stenosis (%) | 8 (1.4) | 6 (2.3) | 0.517 |
| LM stenosis (%) | 1 (0.2) | 0 (0.0) | 1.000 |

*Baseline characteristics of the subgroup of patients with low AHA/ACC PTP ≤15% stratified by CAD-score BMI, body mass index; CACS, coronary artery calcium score, CAD, coronary artery disease; CVD, cardiovascular disease; CX, circumflex artery; IQR, interquartile range; LAD, left anterior descending artery, RCA, right coronary artery; LM, left main; LVEF, left ventricular ejection fraction; QCA, qualitative comparative analysis; SD, standard deviation.*

Table S3 – Baseline characteristics of patients with intermediate-high pre-test probability stratified by low and high CAD-score measurement

|  | CAD-score ≤20 | CAD-score>20 | p-value |
| --- | --- | --- | --- |
| n | 387 | 1657 |  |
| Age, median [IQR] | 51.00 [46.00, 60.08] | 63.00 [57.00, 68.00] | <0.001 |
| Sex, male (%) | 286 (73.9) | 1195 (72.1) | 0.520 |
| Symptoms (%) |  |  | <0.001 |
| Typical chest pain | 89 (23.0) | 462 (27.9) |  |
| Atypical chest pain | 177 (45.7) | 661 (39.9) |  |
| Nonspecific | 107 (27.6) | 382 (23.1) |  |
| Dyspnea | 14 (3.7) | 152 (9.1) |  |
| Smoking (%) |  |  | <0.001 |
| Active smoker | 157 (40.6) | 460 (27.8) |  |
| Former smoker | 92 (23.8) | 394 (23.8) |  |
| Never | 138 (35.7) | 803 (48.5) |  |
| Hypertention (%) | 20 (5.2) | 919 (55.5) | <0.001 |
| RELATI = TRUE (%) | 120 (31.0) | 562 (33.9) | 0.302 |
| Diabetes (%) | 10 (2.6) | 102 (6.2) | 0.008 |
| BMI, mean (SD) | 27.04 (3.81) | 27.41 (4.03) | 0.105 |
| Systolic BP mean (SD) | 126.02 (10.50) | 143.46 (17.34) | <0.001 |
| Diastolic BP, mean (SD) | 79.68 (8.47) | 85.54 (10.22) | <0.001 |
| Heart rate, mean (SD) | 60.98 (9.57) | 62.17 (9.88) | 0.033 |
| LVEF, mean (SD) | 59.90 (2.86) | 59.54 (3.78) | 0.086 |
| AHA/ACC-PTP, median [IQR] | 22.00 [16.00, 32.00] | 32.00 [22.00, 44.00] | <0.001 |
| CAD-score, median [IQR] | 17.00 [15.00, 18.00] | 31.00 [26.00, 39.00] | <0.001 |
| Hemodynynamic obstructive CAD (%) | 365 (94.3) | 1397 (84.3) | <0.001 |
| CACS, median [IQR] | 0.00 [0.00, 18.00] | 33.00 [0.00, 206.00] | <0.001 |
| CACS groups (%) |  |  | <0.001 |
| 1 | 236 (61.1) | 526 (31.8) |  |
| 2 | 137 (35.5) | 866 (52.4) |  |
| 3 | 13 (3.4) | 261 (15.8) |  |
| LAD stenosis (%) | 12 (3.1) | 191 (11.5) | <0.001 |
| RCA stenosis (%) | 7 (1.8) | 127 (7.7) | <0.001 |
| CX stenosis (%) | 4 (1.0) | 118 (7.1) | <0.001 |
| LM stenosis (%) | 3 (0.8) | 10 (0.6) | 0.978 |

*Baseline characteristics of the subgroup of patients with intermediate-high AHA/ACC PTP >15% stratified by CAD-score BMI, body mass index; CACS, coronary artery calcium score, CAD, coronary artery disease; CVD, cardiovascular disease; CX, circumflex artery; IQR, interquartile range; LAD, left anterior descending artery, RCA, right coronary artery; LM, left main; LVEF, left ventricular ejection fraction; QCA, qualitative comparative analysis; SD, standard deviation.*

Table S4 – Diagnostic performance of the CAD-score at cut-off 20 and AHA/ACC-PTP at cut-off 15%

|  | **CAD-score** | **AHA/ACC-PTP** |
| --- | --- | --- |
| Cut-off | 20 | 15% |
| Obstructive CAD, n | 322 | 322 |
| Other (no CAD and non-obstructive CAD), n | 2552 | 2552 |
| Prevalence of CAD (p=0.05566) | 11.2% | 11.2% |
| AUC | 70% (66.7-73.3) | 69.9% (66.6-73.3) |
| NPV | 95.8% (94.4-97%) | 95.2% (93.5-96.5%) |
| PPV | 14.7% (13.2-16.4%) | 13.8% (12.3-15.4%) |
| Sensitivity | 87.6% (83.5-91%) | 87.6% (83.5-91%) |
| Specificity | 35.9% (34.1-37.8%) | 31% (29.2-32.8%) |
| Rule out | 33.3% (31.6-35.1%) | 28.9% (27.2-30.6%) |
| Rule in | 66.7% (64.9-68.4%) | 71.1% (69.4-72.8%) |
| True negative, n | 917 | 790 |
| False negative, n | 40 | 40 |
| False positive, n | 1635 | 1762 |
| True positive, n | 282 | 282 |
| PLR | 1.367 | 1.268 |
| NLR | 0.3457 | 0.4013 |

*AUC, NPV, PPV, sensitivity, specificity, PLR, and NLR for CAD-score at cut-off 20 and AHA/ACC-PTP at cut-off 15%. PLR =* $\frac{sensitivity}{1-specificity}$*, NLR=* $\frac{1-sensitivity}{specificity}$*. AUC, area under the receiver-operating curve; NLR, negative likelihood ratio; NPV, negative predictive value; PLR, positive likelihood ratio; PPV, positive predictive value.*

Table S5: Reclassification table

| Total population | | | | |
| --- | --- | --- | --- | --- |
|  |  | CAD-score | |  |
|  |  | ≤20 | >20 | Total |
| PTP | ≤15% | 570 | 260 | 830 |
|  | >15% | 387 | 1657 | 2044 |
| Total |  | 957 | 1917 | 2874 |
|  |  |  |  |  |
| Patients with hemodynamic obstructive CAD | | | | |
|  |  | CAD-score | |  |
|  |  | ≤20 | >20 | Total |
| PTP | ≤15% | 18 | 22 | 40 |
|  | >15% | 22 | 260 | 282 |
| Total |  | 40 | 282 | 322 |
|  |  |  |  |  |
| Patients without obstructive CAD | | | | |
|  |  | CAD-score | |  |
|  |  | ≤20 | >20 | Total |
| PTP | ≤15% | 552 | 238 | 790 |
|  | >15% | 365 | 1397 | 1762 |
| Total |  | 917 | 1635 | 2552 |
|  |  |  |  |  |
| NRI $\frac{n_{eventcorrectclassification}-n_{eventincorrectclassification}}{n_{event}}+\frac{n_{non-eventcorrectclassification}-n_{non-eventincorrectclassification}}{n_{non-event}}=\frac{22-22}{322}+\frac{365-238}{2552}=0.0498$ | | | | |

*Numbers of patients classified as low and intermediate-high probability of CAD according to the AHA/ACC pre-test probability (PTP) compared to the CAD-score. In patients with obstructive CAD 15 patients were correctly classified upwards to a higher predicted probability, and 5 were incorrectly classified down to a lower probability. In patients without CAD 161 patients were correctly classified to a lower probability group and 160 incorrectly to a higher probability group, yielding a NRI of 0.069. CAD, coronary artery disease; PTP, pre-test probability. Green is correctly reclassified patients. Red is incorrectly reclassified patients.*

Table S6: Study-specific reclassification in low & high PTP.

|  | Dan-NICAD I | | Dan-NICAD II | | Total | |
| --- | --- | --- | --- | --- | --- | --- |
|  | n | % | n | % | n | % |
| n | 1337 |  | 1537 |  | 2874 |  |
|  |  |  |  |  |  |  |
| **PTP >15%** | **878** | **65,7%** | **1166** | **75,9%** | **2044** | **71,1%** |
| CAD-score >20 | 716 | 81,5% | 941 | 80,7% | 1657 | 81,1% |
| CAD-score ≤20 | 162 | 18,5% | 225 | 19,3% | 387 | 18,9% |
| Prevalence of obstructive CAD | 108 | 12,3% | 174 | 14,9% | 282 | 13,8% |
| Prevalence of obstructive CAD & CAD-score >20 | 103 | 14,4% | 157 | 16,7% | 260 | 15,7% |
| Prevalence of obstructive CAD & CAD-score ≤20 | 5 | 3,1% | 17 | 7,6% | 22 | 5,7% |
|  |  |  |  |  |  |  |
| **PTP ≤15%** | **459** | **34,3%** | **371** | **24,1%** | **830** | **28,9%** |
| CAD-score >20 | 133 | 29,0% | 127 | 34,2% | 260 | 31,3% |
| CAD-score ≤20 | 326 | 71,0% | 244 | 65,8% | 570 | 68,7% |
| Prevalence of obstructive CAD | 24 | 5,2% | 16 | 4,3% | 40 | 4,8% |
| Prevalence of obstructive CAD & CAD-score >20 | 11 | 8,3% | 11 | 8,7% | 22 | 8,5% |
| Prevalence of obstructive CAD & CAD-score ≤20 | 13 | 4,0% | 5 | 2,0% | 18 | 3,2% |

**Supplementary figure and figure legend**

## **Figure S1**


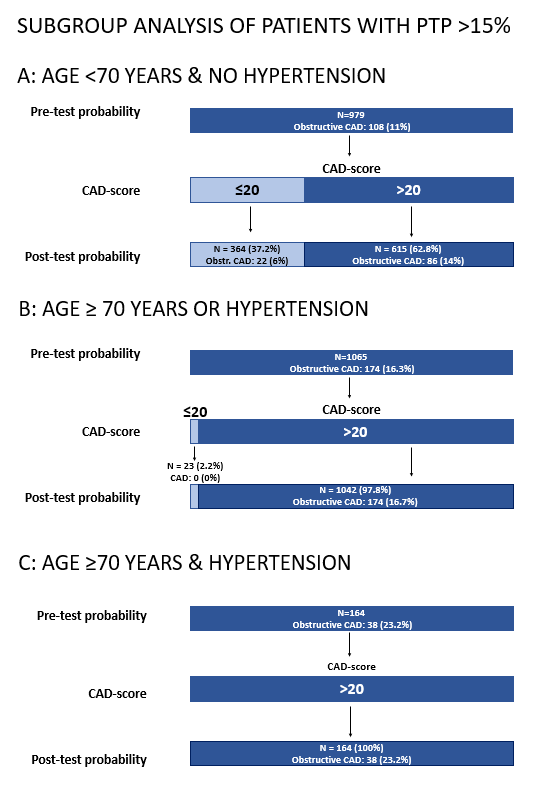


**Figure S1:** Subgroup analysis of reclassification of patients with PTP>15% by CAD-Score in A) patients with age <70, and no hypertension, B) age ≥70 years or hypertension, and C) age ≥70 years and hypertension. CAD, hemodynamically obstructive coronary artery disease; PTP, pre-test probability.
